# Colorectal cancer surgery remains effective with rising patient age

**DOI:** 10.1007/s00384-014-1914-y

**Published:** 2014-06-13

**Authors:** Ulrich Nitsche, Christoph Späth, Tara C. Müller, Matthias Maak, Klaus-Peter Janssen, Dirk Wilhelm, Jörg Kleeff, Franz G. Bader

**Affiliations:** Department of Surgery, Klinikum rechts der Isar, Technische Universität München, Munich, Germany

**Keywords:** Colorectal cancer, Surgery, Age, Old, Elderly

## Abstract

**Background:**

The incidence of colorectal cancer rises disproportionally in aging persons. With a shift towards higher population age in general, an increasing number of older patients require adequate treatment. This study aims to investigate differences between young and elderly patients who undergo resection for colorectal cancer, regarding clinical characteristics, morbidity, and prognosis.

**Methods:**

By retrospective analysis of 6 years (2007 to 2012) of a prospectively documented database, a total of 636 patients were identified who underwent oncological resection for colorectal cancer at our institution. Of this total, all 569 patients with primary colorectal adenocarcinoma were included. Four hundred ten patients were 74 years or younger and 159 were 75 years or older. The median follow-up was 22 months.

**Results:**

Older patients had significantly more comorbidities (85 % vs. 56 %, *p* < 0.001) and a higher ASA score (*p* < 0.001). The mean length of stay in the hospital was longer (24 vs. 20 days, *p* = 0.002), as was the length of postoperative intensive care stay (4 vs. 2 days, *p* = 0.003). However, elderly patients did not have significantly higher rates of intraoperative complications or surgical morbidity. Tumor-specific 2-year survival was 83 ± 4 % for the elderly and 87 ± 2 % for the younger patients, which was not significantly different (*p* = 0.90).

**Conclusions:**

Long-term outcome after oncologic resection for colorectal cancer does not differ between elderly and younger patients. Age in general should not be considered as a limiting factor for colorectal cancer surgery or tumor-specific prognosis.

## Introduction

With a rise from 5 to 14 %, the percentage of people over 80 years is estimated to be more than doubled in 2050 in Western countries [1–3]. Aging may alter the body’s antitumor defenses, making older persons more vulnerable to malignancies [4, 5]. The three main cancer entities whose incidence rates are rising most with advancing population age are prostate, lung, and colorectal cancers [4]. A continuously increasing number of elderly patients with colorectal cancer is seeking treatment, and medical as well as surgical options are improving. Personalized treatment regimens include different neoadjuvant concepts for rectal cancer, limited palliative versus advanced oncological tumor resections, and combinations of chemotherapeutic agents and targeted therapies [6].

Distinct guidelines exist regarding the age of admission to screening programs and stage dependent treatment of colorectal cancer [6–8]. However, in contrast to screening, age is not reflected in treatment specifications [6–8]. Indications or contraindication for neoadjuvant treatment and surgery depending on patient age are not defined in current guidelines at all [7]. Age per se is no contraindication for adjuvant treatment [7], nevertheless, elderly patients are often deprived from advanced systemic [9] or surgical [10] treatment approaches due to comorbidities, supposed compliance, and financial health policy aspects. Special issues include potentially prolonged recovery periods, accumulated comorbidities, and prolongation of quality adjusted life years (QALYs) versus disability-adjusted life years (DALYs). Clinical tools like the ASA score allow estimation of the patient’s individual condition independent of chronological age [1].

Mortality is rather determined by the number of comorbidities than by patient age alone [11]. In fact however, patients aged 75 years have a mean of five comorbidities at the time colorectal cancer is diagnosed [1]. The most relevant comorbidities for surgery are cardiovascular and pulmonary diseases, tumor anemia, and liver or kidney disorders, which are common in elderly patients [1–3]. Furthermore, neurological or psychological disorders and polypharmacy are often prevalent in aged patients [2]. Treatment-related toxicity can occur by differences in the absorption, distribution, metabolism and excretion of drugs [4, 5]. While elderly patients in general are considered to have the same benefit from adjuvant chemotherapy as younger individuals, data from the linked Surveillance, Epidemiology and End Results (SEER)/Medicare database indicate that adjuvant chemotherapy does not substantially improve overall survival in patients over the age of 65 with stage II colon cancer [12].

The only curative treatment option for colorectal cancer remains surgery [6, 10]. Previous studies suggest differences between younger and older patients with colorectal cancer regarding tumor stage, differentiation, and survival [12, 13]. Thus, the indication for colorectal cancer surgery in the elderly is a matter of debate [10]. The question remains whether those patients profit or may even be harmed by extensive surgery [3]. Most prospective clinical trials do not enroll older persons. If they do, individuals are likely to be comorbidity-free, and therefore represent a highly selected group [4, 11]. The aim of this study was to investigate whether age allows stratification into different risk groups. Clinical and survival differences of old versus young patients who all underwent oncological resection for colorectal cancer at a single institution were analyzed.

## Patients and methods

### Patients

All patients undergoing surgery for colorectal cancer between 2007 and 2012 at the Department of Surgery, Klinikum rechts der Isar, Technische Universität München, Munich, Germany, were prospectively documented in a database. The last date of inclusion and follow-up was May 2013. The Institutional Review Board approved prospective data collection and retrospective review of the patient charts for this project. Analysis was conducted on an anonymized data set. Documented data include preoperative performance status, tumor staging and multimodal treatment, details of the surgical procedures, occurrence of complications, postoperative histopathology, application of adjuvant or palliative treatment, and follow-up (date of last visit, date and site of tumor recurrence, date of tumor-related or unrelated death, tumor-specific and recurrence free survival). After discharge of the initial hospitalization, patients are scheduled for periodic follow-up at the interdisciplinary ambulatory tumor center of the clinic or outside the hospital according to the recommendations of the German Cancer Society [7]. The recommendations include regular physical examination, blood analysis, abdomen ultrasonography or computed tomography, chest radiography, and colonoscopy. Information from patients followed extramural is obtained by periodic contacting the responsible general practitioners, gastroenterologists, or patients themselves.

### Statistical analysis

When applicable, age was calculated continuously for comparing young versus elderly patients in order to provide the highest statistical power. For dichotomous group comparisons, all 569 patients were divided into young (<75 years; *n* = 410) and elderly (≥75 years; *n* = 159). Statistical testing was performed using IBM® SPSS® statics Version 19 (SPSS Inc., IBM Corporation Software Group, Somers, NY, USA). The distribution of nominal or ordinal scaled variables was compared by Pearson’s chi-square test. Cardinal variables were tested for normal distribution by visualization on a histogram and by the Kolmogorov–Smirnov test. For comparison of independent groups, the *t* test was used for normal distribution and the Mann–Whitney *U* test for non-normal distribution.

All tests were performed two-sided, and *p* values less than 0.05 were considered to be statistically significant. No correction of *p* values was applied to adjust for multiple test issue. However, the results of all conducted statistical tests are thoroughly reported, so that an informal adjustment of *p* values can be performed while reviewing the data [14].

Time-dependent survival probabilities were estimated with the Kaplan–Meier method and the log-rank test was used to compare subgroups. To investigate the effect on survival of multivariable relationships among covariates, Cox proportional hazard models were used. Survival times as well as estimated hazard ratios (HRs) were calculated and reported in 95 % confidence intervals (CIs). Performed statistical tests are indicated if not self-explanatory.

## Results

### Patient cohort

Between 2007 and 2012, a total of 636 patients underwent surgery for colorectal cancer at our institution. The median follow-up was 22 months (range 1–60 months). In 28 cases, surgery was performed due to recurrence of previous disease. In 39 cases, in situ carcinoma (Tis, UICC 0; *n* = 16) was diagnosed or pathological analysis revealed rare histological entities (adenosquamous carcinoma, gastro-intestinal stroma tumors, neuroendocrine tumor/carcinoma, *n* = 23). Those patients were excluded, leaving finally 569 patients for the analysis.

**Fig. 1:**
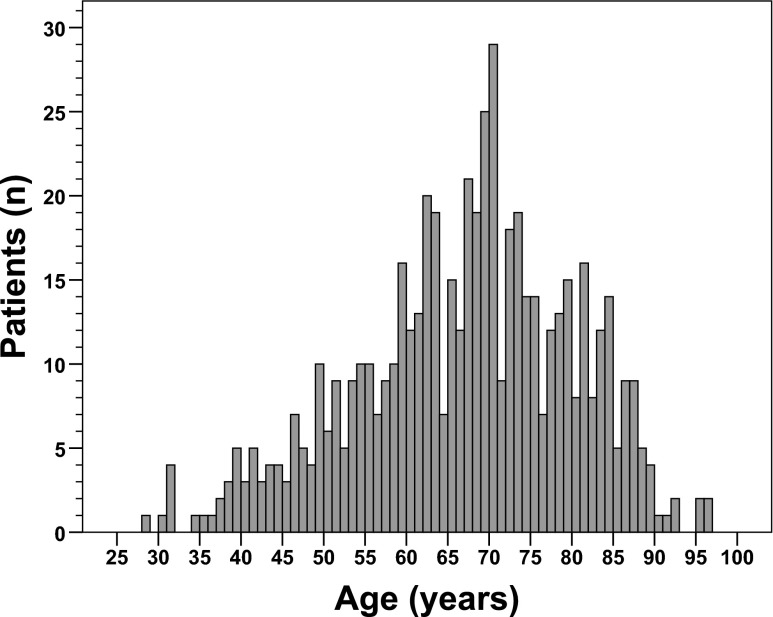
Age distribution of the patient cohort

Of all 569 included patients, there were more men (*n* = 342, 60 %) than women (*n* = 227, 40 %). The mean age was 66 ± 13 years (standard deviation). The median age was 68 years, with the youngest patient being 28 years and the oldest patient being 96 years (Fig. 1). Characteristics of the patient cohort are described in Table 1. The tumor was located in the colon in 65 % (*n* = 371) and in the rectum in 35 % (*n* = 198). Surgical procedures included right hemicolectomy (*n* = 162), resection of the transverse colon (*n* = 5), left hemicolectomy (*n* = 58), resection of the sigmoid (*n* = 81), (sub) total colectomy (*n* = 25), anterior rectal resection (*n* = 182), Hartmann’s procedure (*n* = 22), abdomino-perineal resection (*n* = 29), and transanal endoscopic microsurgery (*n* = 5). The UICC/AJCC tumor stage was stage I in 25 % (*n* = 143), stage II in 26 % (*n* = 149), stage III in 28 % (*n* = 158), and stage IV in 21 % (*n* = 119).

**Table 1:** Clinical and histopathological characteristics of the patient cohort

	Age <75 years *n* = 410 (%)	Age ≥75 years *n* = 159 (%)	All patients *n* = 569 (%)	*p*
Gender				
Male	259 (63)	83 (52)	342 (60)	0.02
Female	151 (37)	76 (48)	227 (40)	
Tumor location				
Coecum — splenic flexure	114 (28)	67 (42)	181(32)	<0.001
Descending colon — sigmoid	137 (55)	55 (35)	192 (34)	
Rectum	159 (37)	37 (23)	196 (34)	
ASA				
I	51 (12)	3 (2)	54 (10)	<0.001
II	254 (62)	66 (42)	320 (56)	
III	77 (19)	77 (48)	154 (27)	
IV	5 (1)	6 (4)	11 (2)	
missing	23 (6)	7 (4)	30 (5)	
Comorbidities				
No	179 (44)	24 (15)	203 (36)	<0.001
Yes	231 (56)	135 (85)	336 (64)	
Tumor obstrucion				
No	318 (78)	124 (78)	442 (78)	0.99
Yes	21 (5)	8 (5)	29 (5)	
Missing	71 (17)	27 (17)	98 (17)	
Presurgical treatment				
No	313 (76)	143 (90)	456 (80)	<0.001
Yes	97 (24)	16 (10)	113 (20)	
Surgery				
Elective	384 (94)	147 (92)	531 (93)	0.61
Emergency	26 (6)	12 (8)	38 (7)	
Surgery: PRBC (mean ± SD)	0.5 ± 1	1 ± 4	0.7 ± 2	0.07
Surgery: duration (min, mean ± SD)	220 ± 90	199 ± 88	214 ± 90	0.02
Multivisceral surgery				
No	335 (82)	135 (85)	47 (83)	0.37
Yes	75 (18)	24 (15)	99 (17)	
Intraoperative complications				
No	397 (97)	150 (94)	547 (96)	0.17
Yes	13 (3)	9 (6)	22 (4)	
General complications				
No	319 (78)	108 (68)	427 (75)	0.02
Yes	91 (22)	51 (32)	142 (25)	
Surgical complications				
No	250 (61)	9 (61)	347 (61)	0.99
Yes	160 (39)	62 (39)	222 (39)	
Anastomotic leakage				
No	394 (96)	15 (96)	546 (96)	0.79
Yes	16 (4)	7 (4)	23 (4)	
Length of hosp. stay (days, mean ± SD)	20 ± 13	24 ± 12	21 ± 12	0.002
Length of ICU stay (days, mean ± SD)	2 ± 3	4 ± 7	2 ± 5	0.003
Perioperative mortality				
No	11 (3)	8 (5)	19 (3)	0.03
Yes	399 (97)	151 (95)	550 (97)	
Postsurgical treatment				
No	233 (57)	120 (76)	353 (62)	<0.001
Chemotherapy	170 (41)	37 (23)	207 (36)	
(Chemo-)radiation	7 (2)	2 (1)	9 (2)	
T				
T1	61 (15)	12 (8)	73 (13)	0.06
T2	72 (18)	26 (16)	98 (17)	
T3	203 (49)	95 (60)	298 (52)	
T4	74 (18)	26 (16)	100 (18)	
N				
N0	222 (54)	100 (63)	322 (57)	0.17
N1	107 (26)	34 (21)	141 (25)	
N2	81 (20)	25 (16)	106 (19)	
Lymph nodes resected (mean ± SD)	19 ± 9	18 ± 7	18 ± 9	0.16
Lymph nodes positive (mean ± SD)	3 ± 5	2 ± 6	2 ± 5	0.47
Lymph node ratio	0.121	0.104	0.117	0.38
M				
M0	314 (77)	136 (86)	450 (79)	0.02
M1	96 (23)	23 (14)	119 (21)	
Stage (UICC/AJCC)				
I	109 (27)	34 (21)	143 (25)	0.004
II	92 (22)	57 (36)	149 (26)	
III	113 (28)	45 (28)	158 (28)	
IV	96 (23)	23 (15)	119 (21)	
Histological grading				
G1/2	273 (67)	117 (74)	390 (68)	0.14
G3/4	132 (32)	42 (26)	174 (31)	
Missing	5 (1)	0	5 (1)	
Lymphatic invasion				
L0	310 (76)	128 (81)	438 (77)	0.21
L1	100 (24)	31 (19)	131 (23)	
Angioinvasion				
V0	359 (88)	140 (88)	499 (88)	0.87
V1	51 (12)	19 (12)	70 (12)	
Perineural invasion				
Pn0	390 (95)	153 (96)	546 (95)	0.57
Pn1	20 (5)	6 (4)	26 (5)	
Tumor type				
Adenocarcinoma	364 (89)	144 (91)	508 (89)	0.42
Mucinous adenocarcinoma	44 (11)	13 (8)	57 (10)	
Signet ring cell carcinoma	2 (0.5)	2 (1)	4 (1)	
R (local)				
R0	381 (93)	151 (95)	532 (93)	0.42
R1	17 (4)	3 (2)	20 (4)	
R2	0	0	0	
RX	12 (3)	5 (3)	17 (3)	
R (systemic)				
R0	336 (82)	141 (89)	477 (84)	0.22
R1	19 (5)	3 (2)	22 (4)	
R2	42 (10)	11 (7)	53 (9)	
RX	13 (3)	4 (2)	17 (3)	

*PRBC* Packed red blood cells, *SD* Standard deviation, *ICU* Intensive care unit

Intraoperative morbidity was 4 % (*n* = 22), including respiratory failure, hemorrhage, or iatrogenic injury of adjacent organs. Postoperative surgical complications occurred in 39 % of all patients (*n* = 222), including minor morbidity like impaired wound healing (*n* = 134) and postoperative intestinal hypomotility or voiding disorder (*n* = 34). The non-surgical morbidity was 25 % (*n* = 142), mainly caused by pneumonia and urinary tract infections. Anastomotic leakage occurred in 4 % (*n* = 23), diagnosed by the drainage fluid, CT scan or surgical re-intervention. The perioperative 30-day mortality was 3 % (*n* = 19), mainly caused by a rapid progress of metastasis with consecutive liver failure or by disseminating infection and septic shock.

The median length of hospital stay was 21 days (range 3–92 days). A total of 374 patients (66 %) were monitored on the intensive care unit postoperatively, with a median stay of 2 days (range 1–47 days). During surgery, packed red blood cells were transfused in 19 % of the patients (109 of all 569 patients). The tumor-specific 2-year survival for all patients was 86 ± 2 %.

### Differences between younger and elderly patients at presentation

All 569 patients were divided into younger (<75 years; *n* = 410) and elderly (≥75 years; *n* = 159). Characteristics of these two groups are depicted in Table 1. There were no significant differences between younger and elderly patients regarding preoperative bowel obstruction by the tumor (*p* = 0.99) or the rate of emergency surgery (*p* = 0.61). However, elderly patients were more likely to have one or more comorbidities (85 % vs. 56 %, *p* < 0.001, chi-square) and had higher ASA scores (ASA III/IV: 52 % vs. 20 %, *p* < 0.001, chi-square). The mean age of patients with ASA I was 54 years (95 % CI 51–58), for ASA II 65 years (63–66), for ASA III 73 years (72–75), and for ASA IV 78 years (70–85; *p* < 0.001 for concordant increase of age and ASA upon one-way ANOVA).

### Perioperative differences between younger and elderly patients

There were no significant differences between younger and elderly patients regarding the frequency of multivisceral resections (*p* = 0.37) or the number of intraoperatively administered packed red blood cells (*p* = 0.07; Table 1). However, more proximal tumors occurred within the elderly (cecum to splenic flexure, 42 % vs. 28 %, *p* < 0.001). There were no significant differences in the distribution of T stage (*p* = 0.06), N stage (*p* = 0.17), tumor grading (*p* = 0.14), mucinous or signet ring cell tumor subtype (*p* = 0.42), lymphovascular invasion (*p* = 0.21), angioinvasion (*p* = 0.87), or perineural invasion (*p* = 0.57; Table 1). Interestingly, elderly patients were less likely to have synchronous distant organ metastasis (14 % vs. 23 %, *p* = 0.02), and thus had lower UICC/AJCC tumor stages (*p* = 0.004). Of note, in spite of the lower tumor stages in elderly patients, the rate of R0 resections did not differ significantly between the two age groups (*p* = 0.42 for the primary colorectal tumor site and *p* = 0.22 for systemic R0 resections).

Elderly patients had a longer stay in the hospital (mean 24 ± 12 vs. 20 ± 13 days, *p* = 0.002, *t* test). Postoperatively, they were more often monitored on the intensive care unit (77 % vs. 62 %, *p* < 0.001). When transferred to the intensive care unit, the stay was longer for elderly patients (mean 4 ± 7 vs. 2 ± 3 days, *p* = 0.003). The number of intraoperative complications did not increase significantly in elderly patients (6 % vs. 3 %, *p* = 0.17), but the mean age for patients developing intraoperative complications was significantly higher (73 vs. 66 years, *p* = 0.02, *t* test). There was no increase of surgical morbidity by age. Elderly patients had the same risk of surgical complications (39 %, *p* = 0.99; Table 1), without relevant changes in the composition. Further, anastomotic leakage occurred in 4 % in both ages groups (*p* = 0.79). The mean age for patients without postoperative surgical complications was 67 years, the mean age for patients with postoperative surgical complications was 66 years (*p* = 0.74, *t* test). In contrast to intraoperative and surgical complications, elderly patients developed more general complications like pneumonia and urinary tract infections (32 % vs. 22 %, *p* = 0.02, chi-square). The mean age for patients without general complications was 66 years, for patients with general complications 69 years (*p* = 0.004, *t* test). High ASA scores were associated with higher levels of general morbidity (*p* < 0.001), but not with intraoperative (*p* = 0.28) or surgical (*p* = 0.28) morbidity, nor with anastomotic leakage (*p* = 0.46). Perioperative mortality within the first 30 days after surgery was significantly elevated in the elderly patient group (5 %, *n* = 8) compared to the younger group (3 %, *n* = 11; *p* = 0.03).

### Differences between younger and elderly patients during follow-up

**Fig. 2:**
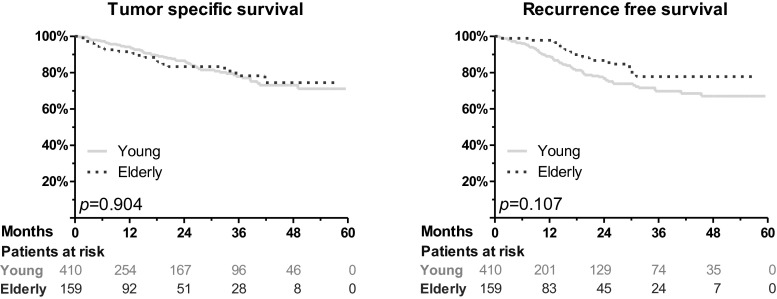
No significant difference in tumor-specific survival and recurrence free survival between young and elderly patients was observed. Apparently, overall survival was reduced for elderly patients (*p* = 0.03; graph not shown)

**Fig. 3:**
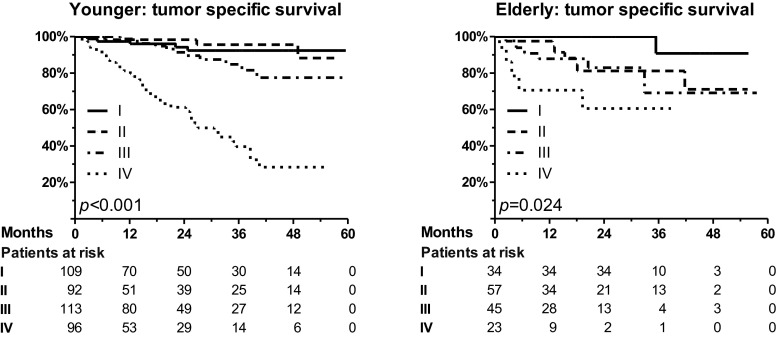
Tumor-specific survival for younger and elderly patients, depending on tumor stage. While the survival was significantly reduced with progressive tumor stages (I, II, III, and IV), a significant difference between younger and elderly patients was only detectable in stage II (see text)

Accompanied by lower tumor stages in elderly patients, the rate of systemic treatment was reduced in this group, both preoperatively (10 % vs. 24 %, *p* < 0.001) and postoperatively (24 % vs. 43 %, *p* < 0.001). However, no significant differences were detected for the rate of tumor recurrence (log rank, young vs. elderly, *p* = 0.11) and tumor-specific survival (*p* = 0.90; Fig. 2). The increase of patient age by 1 year led to a non-significant increase of the risk of tumor-specific death by 1 % (HR 1.01, 95 % CI 0.99–1.03, *p* = 0.21). The tumor-specific 2-year survival for all stages was 87 ± 2 % for the young and 83 ± 4 % for the elderly patients (*p* = 0.90). In particular, for stages I, II, III, and IV, the tumor-specific survival was 94 ± 3 %, 98 ± 2 %, 92 ± 3 %, and 61 ± 6 %, respectively, for the younger patients (Fig. 3). For the elderly patients, it was 100 %, 81 ± 7 %, 83 ± 7 %, and 61 ± 7 %, respectively. Accompanied by small group sizes, there was only a significantly reduced survival for elderly patients in UICC/AJCC stage II (*p* = 0.73 for stage I, p = 0.02 for stage II, *p* = 0.17 for stage III, *p* = 0.96 for stage IV). Finally, age was no independent prognostic factor upon multivariable analysis for tumor-specific survival (Table 2).

**Table 2:** Multivariable analysis of risk factors for tumor-specific survival

	*p*	HR	95 % CI	
			Lower	Upper
Young	1			
vs. elderly	0.79	1.9	0.58	2.05
Men	1			
vs. women	0.03	1.72	1.06	2.79
Colon	1			
vs. rectum	0.86	0.95	0.56	1.62
ASA I	1			
vs. ASA II	0.14	1.98	0.80	4.89
vs. ASA III	0.002	5.01	1.85	13.6
vs. ASA IV	<0.001	49.2	10.8	224
Stage I	1			
vs. stage II	0.40	1.56	0.59	4.34
vs. stage III	0.07	2.48	0.94	6.53
vs. stage IV	0.004	5.06	1.68	15.2
G1/2	1			
vs. G3/4	0.001	2.32	1.39	3.90
R0	1			
vs. R1	0.02	3.65	1.26	10.6
vs. R2	<0.001	4.76	2.07	10.9

Known risk factors like TNM stage and resection status (R) were included in order to test for the independent prognostic capability of patient age (young, 74 years or younger vs. elderly, 75 years or older). Although the hazard ratio (HR) was 1.9-fold elevated for elderly patients, patient age did not turn out to be an independent prognostic factor (*p* = 0.79). In contrast, gender, ASA III/IV, stage III/IV, grading (G), and resection status were independent prognostic factors

*95 % CI* 95 % confidence interval

## Discussion

### Individually tailored treatment regimens and patient age

Multiple treatment options for patients with colorectal cancer exist. Patient age alone does not provide relevant information, rather comprehensive physical assessment is crucial. Biologically younger patients of the same chronological age as prematurely aged patients may require different treatment approaches [1]. The decision whether a patient will profit most from radical surgery or from non-oncological limited procedures in terms of expectancy and quality of life should be made on an individual basis in a multidisciplinary tumor conference. If correctly assessed and treated, the majority of patients older than 80 years maintain social independence after surgery [15]. Although it is difficult to predict the individual outcome of aged multimorbid patients [10], this study shows that oncological long-term results do not differ between age groups.

Here, young versus elderly patients, who all underwent oncological resection for colorectal cancer, were compared. The aim was to reveal relevant differences for elderly patients, who tend to have higher comorbidities. By the retrospective setting, the study presents relevant findings which may not be obtained by prospective trials with strict inclusion criteria regarding comorbidity or age. Only patients undergoing curative or palliative resection of their primary tumor were included in this study. Thus, multimorbid patients who did not undergo surgery were missed. However, inclusion of solely surgical patients allowed the comprehensive analysis of histopathological data, like TNM status and tumor grading.

### Young and elderly patients display specific baseline characteristics

In a review including 34,194 patients from 28 original studies, characteristics of young versus old patients with colorectal cancer with and without tumor resection were analyzed [10]. Preexisting comorbidities were more frequent in the elderly, but it was methodically impossible to perform a meta-analysis due to different definitions and morbidity assessments. Furthermore, complications upon treatment were significantly higher in elderly patients, mainly including respiratory, cardiovascular, cerebrovascular, or thromboembolic morbidity [10]. As found in our study, higher rates of comorbidities and higher ASA scores required a longer hospital stay for elderly patients for the preoperative assessment and postoperative surveillance, as well as longer postsurgical intensive care monitoring. However, an increased number of general complications in elderly patients may also have led to a prolonged hospital and ICU stay [3, 10].

Whether increasing patient age is associated with limited or more advanced tumor stages has been discussed controversially [4, 10, 13]. Some authors describe higher tumor stages for young patients [13], as found in our study. Possible explanations are increased symptom tolerance and less aggressive screening or surveillance among younger people, leading to higher stages at diagnosis. Other authors propagate higher tumor stages for elderly patients, maybe due to masked signs of malignancies and altered presentation of signs and symptoms of cancer in the elderly [4, 10]. Tumor genetics could explain interobserver and interindividual differences [5, 16]. Colorectal cancers of the sporadic microsatellite instability pathway are often associated with advanced patient age, proximal tumor localization, early tumor stages, mucinous or signet ring cell subtype, and poor differentiation but favorable prognosis [13, 17, 18]. This may lead to the reduced rate of synchronous metastasis and the higher rate of right sided tumors that has been identified in the elderly patients of our cohort. Shortening of telomeric length and metabolic changes during cell senescence may play a further not yet identified role [5]. However, neither in this nor in a previous study [18], we identified a correlation of the histological tumor subtype and patient age.

Concordant to our results, the median absolute frequency of postoperative mortality in the studies included in the review mentioned before [10] was 3 % in the less than 65 years age group, 6 % for 65–74 years, 9 % for 75–84 years, and 19 % in the 85 years and above age group. Contradictory to our results, the proportion of patients undergoing emergency surgery was described more than twice as high in the 85 years and above group than in the less than 65 years group [10]. Survival of emergency cases has been shown to be poorer than in those who undergo elective surgery as a result of a higher perioperative mortality rate after emergency surgery (*p* < 0.001 for our cohort, data not shown) [3, 10, 13]. Differences between studies may be explained, at least in part, by the considerably lower number of patients in our analysis.

### Young and elderly patients do not differ in long-term outcome

In concordance with the literature [10], we did not find a significant difference for anastomotic leakage between young and elderly patients. The finding that curative resections decrease with advancing age was not confirmed in our study, however, could be attributed to age-related differences in seeking medical advice, recognition of symptoms, or primary-care referral patterns [10]. In a prospective study of 57 patients aged 80 years or older and undergoing resection of their primary colorectal carcinoma, Kruschewski et al. [3] showed proof that oncological resections should not be denied to older patients. Based on the fact that age-corrected survival of elderly and younger patients is comparable, and in concordance with our study, lethality in elderly patients was identified to be mainly caused by general complications. None of the 57 patients described by Kruschewski et al. [3] died because of surgical morbidity. Thus, limited resections for fear of an otherwise elevated risk of anastomotic leakage cannot be justified [3]. Similarly, O'Connor et al. [12] found that 28 patients aged 40 years and younger had no differences of survival compared to 190 patients aged over 40 years, although younger patients had higher rates of mucinous adenocarcinoma. In contrast, Chou et al. [13] described poorer disease-free survival for elderly patients. However, the latter study raises some questions as older patients with obviously higher chance of dying due to any reason had reduced disease free survival, but not overall survival. The broad literature does not confirm relevant differences of cancer-specific survival between older and younger patients who underwent curative surgery [3, 10]. In a review, which is in line with our data, 2-year tumor-specific survival rates for patients <65, 65–74, 75–85, and ≥85 years were 89 %, 85 %, 85 %, and 78 %, respectively [10].

## Conclusion

Oncological long-term outcome of surgery for colorectal cancer does not differ significantly between elderly and younger patients. Neither intraoperative, nor surgical complications seem to increase with advanced age. However, elderly patients have significantly higher comorbidity. Accurate monitoring is important due to the elevated rate of general morbidity and perioperative mortality in this subgroup.
